# A minimum specification dataset for liquid ocular endotamponades: recommendations by a European expert panel

**DOI:** 10.1007/s00417-023-06289-6

**Published:** 2023-12-01

**Authors:** Mariantonia Ferrara, David HW Steel, Mario R Romano, for the European Intraocular Tamponades Study Group, Aman Chandra, Rosa M Coco-Martin, J Carlos Pastor, Mariantonia Ferrara, Kai Januschowski, Annekatrin Rickmann, Salvador Pastor-Idoate, Mario R Romano, Jonathan Smith, David HW Steel, Martin S Spitzer

**Affiliations:** 1 Manchester Royal Eye Hospital, Oxford Rd, Manchester, M13 9WL UK; 2 Sunderland Eye Infirmary, Sunderland, UK; 3 Bioscience Institute, Newcastle University, Newcastle upon Tyne, UK; 4 Department of Biomedical Sciences, Humanitas University, Pieve Emanuele, Milan, Italy; 5 Department of Ophthalmology, Humanitas Gavazzeni-Castelli, Bergamo, Italy

**Keywords:** Biocompatibility, Ocular endotamponades, Perfluorocarbon liquids, Purity, Safety assessment, Silicone oil

## Abstract

**Purpose:**

To propose a minimum specification dataset to characterize liquid ocular endotamponades (OEs), namely silicone oil (SO), heavy SO (HSO), perfluorodecalin (PFD), and perfluoro-octane (PFO), in terms of physicochemical properties, purity and available evidence of safety, in line with ISO16672:2020.

**Methods:**

An evidence-based consensus using the expert panel technique was conducted. Two facilitators led a committee of 11 European experts. Facilitators prepared a dataset for each compound including the list of specifications relevant for the safety, identified by the group members on the basis of expertise and a comprehensive literature review. Each item was ranked by each member using a 9-point scale from 1 “absolutely to not include” to 9 “absolutely to include” in two rounds followed by discussion. Only items reaching consensus (score ≥ 7 from ≥ 75% of members) were included in the final datasets.

**Results:**

For all OEs, consensus was reached to include manufacturer, density, refractive index, chemical composition, dynamic viscosity, interfacial and surface tension, endotoxins, *in vitro* cytotoxicity assessment, and any evidence from *ex vivo* and/or *in vivo* tests for safety assessment. Additional specifications were added for SO (molecular weight distribution, content of oligosiloxanes with MW ≤ 1000 g/mol, spectral transmittance) and PFD/PFO (% of pure PFD/PFO in the final product, vapor pressure, chemical analyses performed for safety assessment).

**Conclusion:**

The proposed evidence-based minimum specification datasets for SO, HSO, PFD, and PFO have the potential to provide surgeons and health service purchasers with an easily available overview of the most relevant information for the safety assessment of OEs.

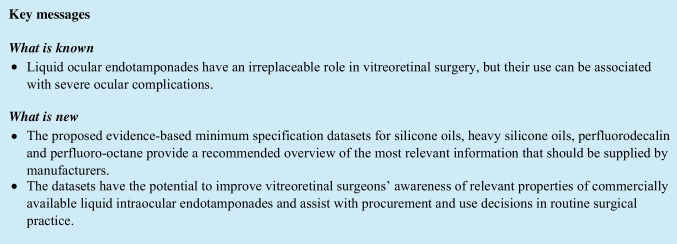

**Supplementary information:**

The online version contains supplementary material available at 10.1007/s00417-023-06289-6.

## Introduction

Liquid ocular endotamponades (OE) are synthetic chemicals, classified as “surgically invasive medical devices” according to the most recent European Union (EU) Regulation 2017/745 on medical devices. In current surgical practice, perfluorodecalin (PFD) and perfluoro-octane (PFO), belonging to the class of perfluorocarbon liquids (PFCL), are mainly used intraoperatively to flatten and stabilize detached retinas and facilitate surgical maneuvers [1–4], whereas silicone oils (SO), polymers of polydimethylsiloxane, and heavy SO (HSO), mixtures of SO and semi-fluorinated alkanes, are used as long-term endotamponades in various complex vitreoretinal pathologies, including retinal detachments [5]. Although so far irreplaceable, these compounds can lead to severe ocular complications [1, 6]. Emulsification is known to play a major role in SO- and HSO-related complications [1, 6]. Furthermore, the presence of contaminants is a recognized critical issue of liquid OE, in particular low molecular weight components (LMWC), short-chain siloxanes, in SO, and incompletely fluorinated contaminants and oxygen-containing compounds in PFCL [7].

In order to be approved for surgical use within the European Union, OE need to conform to the directives for the Conformité Européene (CE) marking, the requirements formulated in the Medical Device Regulation 2017/745 and specific International Organization for Standardization (ISO) standards. Nevertheless, the recent report of cases of severe acute ocular toxicity after the intraoperative use of a PFO certified as safe has raised significant concerns among the scientific community on the adequacy of the current regulations and testing methods [8, 9]. In this regard, recent laboratory studies are providing growing evidence on the physicochemical characterization of PFCL and SO, validation of testing protocols, the nature and potential cytotoxicity of contaminants, and corresponding toxicity thresholds [7, 10–23]. Furthermore, significant variability in the composition and purity profile of commercially available SO and PFCLs has been demonstrated [21, 22], highlighting the lack of fundamental information for vitreoretinal surgeons in order to critically and consciously evaluate the findings of different products and reliably compare them to each other [24].

In this light, the aim of this initiative was to propose a minimum specification dataset for the characterization of the OE in terms of their physicochemical properties, purity, and available evidence of safety, in line with ISO16672:2020 [7]. These datasets may raise awareness among vitreoretinal surgeons about the properties of these devices and lead to a more informed choice in their practice.

## Methods

The nominal group or expert panel technique was used to conduct this consensus [25].

A European intraocular tamponade study group consisting of a group of vitreoretinal surgeons with relevant clinical experience and involved in research on OEs, led by two facilitators (MRR and DHWS), was used to carry out the project. Based on their expertise and a comprehensive literature review, the group members defined a comprehensive list of attributes important for the safe and optimal use of SO, HSO, and PFCLs. Recommended values for a certain attribute were included if it was felt that these should be supplied to vitreoretinal surgeons to provide a reliable overview of the quality and safety of a specific OE product before use. For some of the OE characteristics, a specific safety cutoff value did not exist; and in these cases, recommended values were derived based on a literature review where a cutoff was considered relevant for safety. The facilitators summarized the proposals in a dataset draft for each class of OE, including references of key publications. A questionnaire was prepared to evaluate the appropriateness of each attribute to be included in the dataset and any corresponding recommended safety values proposed (Supplementary Table 1–3). Each participant ranked each attribute according to a 9-point scale from 1 “absolutely no” to 9 “absolutely yes” [25]. Facilitators tabulated the rankings for the discussion. Attributes and recommended values ranked between 7 and 9 by at least 75% of the group members were included in the final proposal as consensus was achieved. Ranked ideas were discussed and reranked in order to obtain the final proposal. Attributes and cutoff ranked less between 1 and 3 by at least 75% of the group members were excluded.

Based on the results of the first ranking and associated discussion, a second proposal of datasets was circulated among the group members and reranked. Items that reached consensus were included in the final datasets.

## Results

The final datasets are shown in Tables 1, 2, and 3. These datasets can be applied to any commercially available SO, HSO, PFD, and PFO for ophthalmic use.

**Table 1:** Minimum specification dataset for silicone oils

	Technical information	Recommended values	Comments
1	Manufacturer		
2	Density (g/cm^3^ at 25 °C)		Typically, 0.967–0.975 g/ml at 25 °C
3	Refractive index		Typically, 1.4013–1.4055; using a refractometer at 35 ± 2 °C and 546 ± 10 nm or 589 ± 10 nm wavelength (according to ISO16672:2020)
4	Is the final oil a mixture of two or more compounds of different molecular MW? If yes, specify the compounds		
5	Molecular weight distribution, expressed as polydispersity index (M_w_/M_n_). Please, specify the method used	≤ 2	The average molecular mass, the range of molecular mass distribution and the polydispersity shall be reported according to ISO 16672:2020. We have chosen to use the polydispersity index Mw/Mn as computed by 10.1167/tvst.8.5.9
6	Dynamic viscosity (mPa·s)		At (35 ± 2) °C in the frequency range between 0.01 s–1 and 100 s–1 (according to ISO16672:2020)
7	Interfacial tension (mN/m)		At (35 ± 2) °C (according to ISO16672:2020)
8	Surface tension (mN/m)		At (35 ± 2) °C (according to ISO16672:2020)
9	Spectral transmittance		Measured by transmission spectrophotometry over the range 300 to 1100 nm (according to ISO16672:2020)
10	Content of LMWC with MW ≤ 1000 g/mol Please, specify the method used	Target < 100 ppm	Potential to act as emulsifier. Demonstrated ability to penetrate into retinal tissue. May be responsible of long-term toxicity (https://echa.europa.eu/it/candidate-list-table/-/dislist/details, Nakamura K, et al. Invest Ophthalmol Vis Sci 1991:32,3007-20
11	Endotoxins	≤ 0.2 EU/mL	According to ISO16672:2020
12	*In vitro* cytotoxicity assessment Please, specify (1) method used; (2) testing conditions (cell lines, culture medium, area of contact, time of contact, number of replicates); (3) methodology validation; (4) qualitative assessment; (5) quantitative assessment		According to ISO 10993:5 (reactivity grade < 2; cellular viability > 70%) The use of direct contact test with both BALB 3T3 and ARPE-19 has been validated for in vitro cytotoxicity test for SO. However, ARPE-19 cells have been selected as the most appropriate cell line because these cells showed higher sensitivity in cytotoxicity testing. Moreover, these cells mimic the cells in clinical use (10.1016/j.exer.2020.108018)
13	Any evidence from *ex vivo* and/or *in vivo* tests for safety assessment? If yes, please provide the bibliographic reference(s)		

1) The intended surgical use and any contraindications (e.g. direct exchange with PFCLs)

2) The recommended maximum intraocular retention (months)

3) Recommended shelf-life (months)

*EU* endotoxin units, *LMWC* low molecular weight components, *MW* molecular weight

*doi: 10.1016/j.exer.2020.108018

**Table 2:** Minimum specification dataset for heavy silicone oils

	Technical information	Recommended values	Comments
1	Manufacturer		
2	Density (g/cm^3^ at 25 °C)		Typically, 1.02–1.06 g/ml at 25 °C*
3	Refractive index		Typically, 1.387–1.4* Using a refractometer at 35 ± 2 °C and 546 ± 10 nm or 589 ± 10 nm wavelength (ISO16672:2020)
4	Is the final oil a mixture of two or more compounds of different structure and/or molecular MW? If yes, specify the compounds		
5	Dynamic viscosity (mPa·s)		At (35 ± 2) °C in the frequency range between 0.01 s–1 and 100 s–1, according to ISO16672:2020
6	Interfacial tension (mN/m)		
7	Surface tension (mN/m)		
8	Spectral transmittance		Measured by transmission spectrophotometry over the range 300 to 1100 nm (according to ISO16672:2020)
	Content of LMWC with MW ≤ 1000 g/mol. Please, specify the method used	Target < 100 ppm	Potential to act as emulsifier. Demonstrated ability to penetrate into retinal tissue. May be responsible of long-term toxicity (https://echa.europa.eu/it/candidate-list-table/-/dislist/details, Nakamura K, et al. Invest Ophthalmol Vis Sci 1991:32,3007-20
10	Endotoxins	≤ 0.2 EU/mL	According to ISO16672:2020
11	*In vitro* cytotoxicity assessment. Please, specify (1) method used; (2) testing conditions (cell lines, culture medium, area of contact, time of contact, number of replicates); (3) methodology validation; (4) qualitative assessment; (5) quantitative assessment		According to ISO 10993:5 (reactivity grade < 2; cellular viability > 70%) The use of direct contact test with both BALB 3T3 and ARPE-19 has been validated for in vitro cytotoxicity test for SO. However, ARPE-19 cells have been selected as the most appropriate cell line because these cells showed higher sensitivity in cytotoxicity testing. Moreover, these cells mimic the cells in clinical use (10.1016/j.exer.2020.108018)
12	Any evidence from *ex vivo* and/or *in vivo* tests for safety assessment? If yes, please provide the bibliographic reference(s)		

1) The intended surgical use and any contraindications (e.g. direct exchange with PFCLs)

2) The recommended maximum intraocular retention (months)

3) Recommended shelf-life (months)

*EU* endotoxin units, *LMWC* low molecular weight components, *MW* molecular weight

*variation related to the specific type of HSO

**Table 3:** Minimum specification dataset for perfluorodecalin and perfluoro-octane

	Technical information	Recommended values	Comments
1	Manufacturer		
2	% of pure PFCL in the final product		PFO ≥ 99.0%; PFD ≥ 97.0%
3	Density (g/cm^3^ at 25 °C)		Typically, 1.76–2.03 g/ml at 25 °C*
4	Refractive index		Typically, 1.27–1.33* Using a refractometer at 35 ± 2 °C and 546 ± 10 nm or 589 ± 10 nm wavelength (ISO16672:2020)
6	Interfacial tension (mN/m)		
7	Surface tension (mN/m)		
8	Vapor pressure (mmHg – mbar) at 25 and 37 °C		doi: 10.1038/s41433-022-02021-6
9	Endotoxins	≤ 0.2 EU/mL	According to ISO16672:2020
10	Chemical analyses performed for safety assessment - Number and type of analyses performed - H-value (ppm) - Content of known contaminants (ppm)	< 10 ppm	H-value is a parameter suggested to assess the amount of reactive, underfluorinated compounds and their degradation products (ISO16672:2020)
11	*In vitro* cytotoxicity assessment Please, specify (1) method used; (2) testing conditions (cell lines, culture medium, area of contact, time of contact, number of replicates); (3) methodology validation; (4) qualitative assessment; (5) quantitative assessment		According to ISO 10993:5 (reactivity grade < 2; cellular viability > 70%) The use of direct contact test with both BALB 3T3, ARPE-19, and L929 has been validated for in vitro cytotoxicity test. However, ARPE-19 cells have been selected as the most appropriate cell line because these cells showed higher sensitivity in cytotoxicity testing. Moreover, these cells mimic the cells in clinical use**
12	Any evidence from *ex vivo* and/or *in vivo* tests for safety assessment? If yes, please provide the bibliographic reference(s)		

1) The intended surgical use and any contraindications (e.g., use with silicone oil)

3) Recommended shelf life (months)

*EU* endotoxin units, *PFD* perfluorodecalin, *PFO* perfluoro-n-octane

*variation related to the specific type of PFCL

**10.1167/tvst.8.5.24; 10.1038/s41598-018-19428-5; 10.1021/acsomega.2c04697

### Silicone oils

The specifications that reached absolute consensus after the first ranking were the following: manufacturer, molecular weight distribution expressed as polydispersity index, content of oligosiloxanes with molecular weight (MW) ≤ 1000 g/mol, endotoxin content (according to the limits imposed by ISO16672:2020), and details of *in vitro* cytotoxicity assessment.

After first round discussion and second ranking, the following specifications reached consensus for inclusion: density, refractive index, specification of different compounds (in case of final oil being a mixture of two or more compounds of different molecular MW), dynamic viscosity, interfacial tension, surface tension, spectral transmittance, and details on other biological analyses, namely *ex vivo* and/or *in vivo* tests. In addition, two recommended cut-offs were identified:

- ≤ 2 for polydispersity index
- < 100 parts per million (ppm) for the content of LMWC with MW ≤ 1000 g/mol

### Heavy silicone oils

Absolute consensus was achieved after the first ranking for the following specifications: manufacturer, density, content of oligosiloxanes with MW ≤ 1000 g/mol, endotoxin content (according to the limits imposed by ISO16672:2020), and details of *in vitro* cytotoxicity assessment.

The specifications that were discussed and reached consensus at the second ranking were the following: refractive index, specification of different compounds (in the case of silicone oil component was a mixture of two main PDMS polymers of different molecular MW), dynamic viscosity, interfacial tension, surface tension, spectral transmittance, and details on other biological analyses, namely *ex vivo* and/or *in vivo* tests. After discussion, a value of < 100 ppm was recommended as cutoff for the LMWC with MW ≤ 1000 g/mol content.

### Perfluorodecalin and perfluoro-octane

After the first round of ranking, the following specifications achieved consensus: manufacturer, percentage of pure compound (PFD or PFO), density, content of endotoxin (according to ISO16672:2020), H-value (with a cutoff of < 10 ppm), and details of *in vitro* cytotoxicity assessment.

After discussion and second ranking, the following specifications were added to the final dataset: refractive index, interfacial tension, surface tension, vapor pressure, details of chemical analyses other than evaluation of H-value, and details of other biological analyses.

## Discussion

The biocompatibility of OEs, related to their intended intraocular use, is a critical issue in vitreoretinal surgery. There is currently an active discussion in the scientific community about the evaluation of purity and safety of OE [8, 9, 24]. In particular, it has been highlighted that the information currently provided by manufacturers on their composition is limited, making it impossible for surgeons to reliably compare the products available [24]. With these limitations, the evidence provided by experimental studies plays a crucial role to better characterize these products and their safety profiles [10–23].

As highlighted by ISO 16672:2020 [7], the detection of potentially hazardous contaminants in OEs is another critical issue for their quality and safety evaluation. Related to this, we included in the proposed dataset specifications for the contaminants most commonly detected in PFCLs, including reactive under-fluorinated compounds and other specific known contaminants, and impurities for SO, such as LMWCs. The need to perform chemical analyses or biological analyses or both in order to properly assess the potential cytotoxicity of these compounds has been debated. Chang and Simpson stated that physicochemical analytical techniques were sufficient to detect and measure the concentration of PFCL contaminants [26]; however, it has been pointed out that only biological analyses can effectively demonstrate if certain compounds at certain concentrations exert a toxic effect or not [12, 14, 18]. A combination of physicochemical and biological analyses would appear to be the most preferable, allowing a comprehensive assessment of the safety profile of any particular OE. In this regard, *in vitro* cytotoxicity tests have an established primary role in the assessment of the safety profile of intraocular medical devices [27]. In particular, direct contact cytotoxicity tests have been validated as appropriate and reliable testing method for all the liquid OE, and ARPE-19 cells have been selected as the most appropriate cell line because these cells showed higher sensitivity in cytotoxicity testing and mimic the cells in clinical use [10, 14–16, 23]. The evidence from the literature was not judged enough to make any further specific recommendations.

With the aim of providing vitreoretinal surgeons with a concise overview of relevant information on PFCL, SO, and HSO, we propose three minimum specification datasets, in line with ISO16672:2020 [7]. Indeed, these datasets would summarize the main physicochemical properties, purity and safety of liquid OEs, facilitating a more informed choice by surgeons with up-to-date available guidance for their characterization and biocompatibility. For all the compounds, some brief introductory information on intended surgical use, potential contraindications (e.g., direct exchange with PFCLs), recommended maximum intraocular retention, and shelf life have been added in combination with the dataset for matter of completeness.

With regard to SO, the specifications for which a cutoff was not established by the current regulations were molecular weight distribution (MWD) and LMWC content. The rationale of the inclusion of such findings and the recommended values were agreed based on the available literature.

We proposed a cutoff of ≤ 2 for the polydispersity index of SO. The synthesis of SO leads to the formation of siloxane chains of different length and, despite subsequent purification and ultra-purification processes, the final SO is a mixture of a dominant fraction, made up of polymers of the desired degree of polymerization, and siloxanes chains of different lengths (and thus MW) [28]. It follows that for each final product, a certain MWD can be measured. A broad MWD reflects the presence of compounds of undesired MW, including oligosiloxanes and short-chain siloxane polymers, termed “low molecular weight components” (LMWC) and recognized as impurities. On the other hand, a narrow MWD indicates a higher degree of purity of the final SO. Methodological variations can lead to MW averages not comparable between different laboratories, whereas polydispersity indexes are less influenced by these differences and, thus, can be used as a more reliable marker of SO purity [22]. In particular, we have chosen the polydispersity index resulting from the ratio between the weight average molecular weight (Mw) and numeric molecular weight average (Mn), that has been applied in previous publications on SO for ophthalmic use [21, 22]. The recommended value of ≤ 2 has been proposed as shown to be an achievable level for SO of different nominal viscosities [21, 22].

A value of less than 100 ppm has been recommended as cutoff for LMWC with MW ≤ 1000 g/mol. Several safety concerns are associated with these synthesis-related impurities, such as their ability to diffuse into the ocular tissues, to act as emulsifier for SO, and to induce severe intraocular inflammatory reactions [29, 30]. In addition, octamethylcyclotetrasiloxane (D4), decamethylcyclopentasiloxane (D5), and dodecamethylcyclohexasiloxane (D6) have been added to the Candidate List of Substances of Very High Concern for authorization by the European Chemicals Agency because of their tissue persistence, bioaccumulation, and toxicity (https://echa.europa.eu/it/-/ten-new-substances-added-to-the-candidate-list). It has been recently demonstrated that pure samples of hexamethyldisiloxane (L2), octamethyltrisiloxane (L3), decamethyltetrasiloxane (L4), D4 and D5 can exert an acute cytotoxic effect on retinal cells in vitro [31]. Although no acute cytotoxicity has been found exposing both ARPE-19 and BALB3T3 cells for 24-h to a concentrate of LMWC corresponding to the amount distilled from the SO bulk (and thus at a clinically relevant concentration), detrimental/toxic effects cannot be ruled out in the long-term [16]. As the efficiency of purification and ultra-purification processes decreases as the MW of the siloxane chain increases [22], it appears likely that a certain amount of LMWC of higher MW will be present in the final SO, despite the aim to achieve the lowest possible content. The content of LMWC with MW ≤ 1000 g/mol in 10 different commercially available SO has been previously analyzed, and the lowest values achieved were 51 and 90 ppm [21]. In view of this, we suggested a cutoff of < 100 ppm as the recommended value for LMWC with M ≤ 1000 g/mol for both SO and HSO. It should be observed that due to the current limited knowledge in terms of characterization of HSOs, the data available for SO has been taken as reference.

Similar to SO and HSO, also for PFD and PFO, the rationale for recommending the cutoffs was based on the available literature when an existing criterion had not been set by the current regulations, such as for the content of pure PFD/PFO in the final product, H-value, and content of known contaminants. In general, the synthesis of PFCL is accompanied by the formation of by-products and reactive, incompletely fluorinated compounds, that are known to exert toxic effects [7, 32]. As for SO, the aim of the subsequent purification and ultra-purification processes is to obtain final products with the lowest possible concentration of such contaminants [7].

The values proposed of 99% for PFO and 97% for PFD have been demonstrated to be achievable, as shown in [19]. The difference in the cutoff recommended for PFD and PFO relates to the higher formation of stable fully fluorinated by-products during PFD synthesis [19]. Although we have recommended values so that surgeons can be confident in the composition of what they are using, the measurement of the concentration of the main PFCL component appeared to have limited value in the assessment of the quality of the final product as PFCLs with a similar percentage of pure main component can vary significantly in terms of other contaminants content [19].

The term “H-value” indicates the content (in ppm) of reactive partially hydrogenated perfluoroalkanes, measured through the electrochemical quantification of fluorine ions originating from the reaction of these compounds with hexamethylenediamine [20]. As measured for partially hydrogenated perfluoroalkanes, the H-value has been proposed as safety parameter for PFCL and the cutoff of 10 ppm (detection limit) as safety threshold [20]. Nevertheless, the appropriateness of this parameter to ensure the absence of cytotoxicity of PFCL is currently under debate [18, 33, 34]. In this regard, it has been highlighted that the analytical method to determine the H-value is able to detect a limited range of partially fluorinated compounds as the presence of a CHF−CF2 moiety is required for the reaction to happen, and this moiety is not present in all the compounds identified so far as toxic contaminants of PFCL [18, 33]. In addition, Ruzza et al [18] have reported that the H-content of known PFCL contaminants, such as perfluoro-octanoic acid (PFOA), does not correlate with their cytotoxicity and then, cannot be used as unique parameter to assess PFCL safety. This led us to highlight the importance of combining chemical analyses and adding this specification in the dataset. Recent experimental studies investigating various toxic batches of PFCL have identified a large range of contaminants proposed to be responsible for toxicity, shown in Table 4 [8, 11, 12, 35]. Once detected as contaminants through chemical analyses, some of these compounds have been tested through *in vitro* cytotoxicity tests in order to confirm their cytotoxic effect [ 8,11,12,35]. Several concentrations have been tested through direct contact *in vitro* cytotoxicity tests in order to detect the minimal cytotoxic concentration, derive dose-response curves, and calculate the cytotoxic concentration (CC30) [14].

It is worth noting that the mechanisms of OE-related complications are still not fully understood [1] and this is an intrinsic limitation of the proposed datasets as some relevant factors may have been overlooked. For instance, recent experimental studies have suggested that interactions between different compounds used routinely during surgery may mediate their combined effects on retinal cell viability [17, 36]. Further investigations are required, and this aspect has not been included in the datasets but may need to be added as further evidence emerges.

In conclusion, proposed minimum specification datasets for SO, HSO, and PFCL have been composed using an evidence-based approach to succinctly summarize the most relevant information for the safety assessment of OEs by surgeons and health service purchasers. It is hoped that these will be completed by manufacturers and mandated by procuring authorities.

### Supplementary information

ESM 1 (DOCX 18 kb)

ESM 2 (DOCX 16 kb)

ESM 3 (DOCX 17 kb)
