# Correction: A minimum specification dataset for liquid ocular endotamponades: recommendations by a European expert panel

**DOI:** 10.1007/s00417-024-06369-1

**Published:** 2024-01-09

**Authors:** Mariantonia Ferrara, David H. W. Steel, Mario R. Romano, for the European Intraocular Tamponades Study Group, Aman Chandra, Rosa M. Coco-Martin, J. Carlos Pastor, Mariantonia Ferrara, Kai Januschowski, Annekatrin Rickmann, Salvador Pastor-Idoate, Mario R. Romano, Jonathan Smith, David H. W. Steel, Martin S. Spitzer

**Affiliations:** 1 Manchester Royal Eye Hospital, Oxford Rd, Manchester, M13 9WL UK; 2 Sunderland Eye Infirmary, Sunderland, UK; 3 Bioscience Institute, Newcastle University, Newcastle Upon Tyne, UK; 4 Department of Biomedical Sciences, Humanitas University, Pieve Emanuele, Milan Italy; 5 Department of Ophthalmology, Humanitas Gavazzeni-Castelli, Bergamo, Italy

**Correction: Graefe's Archive for Clinical and Experimental Ophthalmology**

10.1007/s00417-023-06289-6

In the published version of the original paper, the collaborator names were incorrectly captured after the closing of the institutional author resulting to incorrect output of the online version. All the collaborator names should be captured within the institutional author. The PDF shows the correct output.

The original article has been corrected.

