# Supplementary material for: A minimum specification dataset for liquid ocular endotamponades: recommendations by a European expert panel
Source: Graefes Arch Clin Exp Ophthalmol. 2023 Dec 1;262(4):1141–9. doi: 10.1007/s00417-023-06289-6 (PMC10995036; doi:10.1007/s00417-023-06289-6)
Supplement: Supplementary file 1 — (DOCX 18 kb) [file 417_2023_6289_MOESM1_ESM.docx]

**Supplementary Table 1.** Minimum specification dataset for silicone oils questionnaire

|  | **Attribute** | **Score from 1 “absolutely no” to 9 “absolutely yes”** | **Free comment** |
| --- | --- | --- | --- |
| 1 | Manufacturer |  |  |
| 2 | Density (g/cm^3^ at 25°C) |  |  |
| 3 | Refractive index |  |  |
| 4 | Specification of different compounds if the final oil a mixture of two or more compounds of different molecular MW? |  |  |
| 5 | Molecular mass distribution, expressed as polydispersity index (M_w_/M_n_)   - If yes, would you indicate any cutoff? - If yes, what cutoff? - Is it relevant to specify the methodology? |  |  |
|  |  |  |  |
|  |  |  |  |
|  |  |  |  |
| 6 | Dynamic viscosity (mPa·s)   - If yes, would you indicate any cutoff? - If yes, what cutoff? |  |  |
|  |  |  |  |
|  |  |  |  |
| 7 | Interfacial tension (mN/m) |  |  |
| 8 | Surface tension (mN/m) |  |  |
|  | Spectral transmittance |  |  |
| 9 | Vapour pressure (mmHg) |  |  |
| 10 | Oligosiloxanes content with MW ≤ 1,000 g/mol   - If yes, would you indicate any cutoff for components up to MW ≤ 1,000 g/mol? - Is it relevant to specify the methodology? |  |  |
|  |  |  |  |
|  |  |  |  |
| 11 | Endotoxin (according to ISO16672:2020) |  |  |
| 12 | Total level EO and ECH (according to ISO16672:2020) |  |  |
| 13 | In vitro cytotoxicity assessment   - Is it relevant to specify the methodology? |  |  |
|  |  |  |  |
| 14 | Other available biological analyses (ex vivo and /or in vivo tests) for dìsafety assessment |  |  |
