# Supplementary material for: A minimum specification dataset for liquid ocular endotamponades: recommendations by a European expert panel
Source: Graefes Arch Clin Exp Ophthalmol. 2023 Dec 1;262(4):1141–9. doi: 10.1007/s00417-023-06289-6 (PMC10995036; doi:10.1007/s00417-023-06289-6)
Supplement: Supplementary file 2 — (DOCX 16 kb) [file 417_2023_6289_MOESM2_ESM.docx]

**Supplementary Table 2.** Minimum specification dataset for heavy silicone oils questionnaire

|  | **Attribute** | **Score from 1 “absolutely no” to 9 “absolutely yes”** | **Free comment** |
| --- | --- | --- | --- |
| 1 | Manufacturer |  |  |
| 2 | Density |  |  |
| 3 | Refractive index |  |  |
| 4 | Specification of different compounds |  |  |
| 5 | Molecular mass distribution   - If yes, would you indicate any cutoff? - If yes, what cutoff? |  |  |
|  |  |  |  |
|  |  |  |  |
| 6 | Dynamic viscosity   - If yes, would you indicate any cutoff? - If yes, what cutoff? |  |  |
|  |  |  |  |
|  |  |  |  |
| 7 | Interfacial tension |  |  |
| 8 | Surface tension |  |  |
| 9 | Vapour pressure |  |  |
| 10 | Oligosiloxanes content   - If yes, would you indicate any cutoff for components up to MW ≤ 1,000 g/mol? |  |  |
|  |  |  |  |
| 11 | Endotoxin (according to ISO16672:2020) |  |  |
| 12 | Total level EO and ECH (according to ISO16672:2020) |  |  |
| 13 | Biological analyses |  |  |
| 14 | In vitro cytotoxicity assessment |  |  |
