# Supplementary material for: A minimum specification dataset for liquid ocular endotamponades: recommendations by a European expert panel
Source: Graefes Arch Clin Exp Ophthalmol. 2023 Dec 1;262(4):1141–9. doi: 10.1007/s00417-023-06289-6 (PMC10995036; doi:10.1007/s00417-023-06289-6)
Supplement: Supplementary file 3 — (DOCX 17 kb) [file 417_2023_6289_MOESM3_ESM.docx]

**Supplementary Table 3.** Minimum specification dataset for perfluoro-octane/perfluorodecalin questionnaire

|  | **Attribute** | **Score from 1 “absolutely no” to 9 “absolutely yes”** | **Free comment** |
| --- | --- | --- | --- |
| 1 | Manufacturer |  |  |
| 2 | Percentage of pure PFD / PFO in the final procduct |  |  |
| 3 | Density |  |  |
| 4 | Refractive index |  |  |
| 5 | Dynamic viscosity   - If yes, would you indicate any cutoff? - If yes, what cutoff? |  |  |
|  |  |  |  |
|  |  |  |  |
| 6 | Interfacial tension |  |  |
| 7 | Surface tension |  |  |
| 8 | Vapour pressure |  |  |
| 9 | Oligosiloxanes content   - If yes, would you indicate any cutoff for components up to MW ≤ 1,000 g/mol? |  |  |
|  |  |  |  |
| 10 | Endotoxin (according to ISO16672:2020) |  |  |
| 11 | Total level EO and ECH (according to ISO16672:2020) |  |  |
| 12 | Chemical analyses |  |  |
| 13 | H-value   - If yes, cut-off <10 ppm? |  |  |
| 14 | Content of known contaminants |  |  |
| 16 | In vitro cytotoxicity assessment |  |  |
